# Inside-out: modelling the link between Zika virus viral dynamics within hosts and transmission to vectors across host species and virus strains

**DOI:** 10.1098/rsif.2025.0365

**Published:** 2025-10-01

**Authors:** Hélène Cecilia, Benjamin M. Althouse, Sasha R. Azar, Shannan L. Rossi, Nikos Vasilakis, Kathryn A. Hanley

**Affiliations:** ^1^ Department of Biology, New Mexico State University, Las Cruces, NM 88003, USA; ^2^ Oniris, INRAE, BIOEPAR, 44300 Nantes, France; ^3^ Information School, Univerisity of Washington, Seattle, WA 98105, USA; ^4^ Department of Pathology, The University of Texas Medical Branch at Galveston, Galveston, TX 77555-0609, USA; ^5^ Center for Tissue Engineering, Department of Surgery, Houston Methodist Hospital, Houston, TX 77030, USA; ^6^ Department of Microbiology and Immunology, The University of Texas Medical Branch at Galveston, Galveston, TX 77555-0609, USA; ^7^ Center for Vector-Borne and Zoonotic Diseases, The University of Texas Medical Branch at Galveston, Galveston, TX 77555-0609, USA; ^8^ Institute for Human Infection and Immunity, The University of Texas Medical Branch at Galveston, Galveston, TX 77555-0610, USA

**Keywords:** phenomenological model, Zika virus, within-host, vector competence, infectiousness

## Abstract

Epidemiological models of mosquito-borne virus transmission often lack accurate estimates of host-to-vector transmission probability. Here, we estimated this probability for two strains of Zika virus (ZIKV)—one sylvatic and one human-endemic—from two monkey species to *Aedes albopictus* mosquitoes using experimental infection data. Viral dynamics did not differ between monkey species, although one (cynomolgus macaque) is a native ZIKV host and the other (squirrel monkey) a novel host, but did differ between strains, with viremia for the human-endemic strain peaking later and lower than the sylvatic strain. Only the sylvatic strain was transmitted to mosquitoes. Within mosquitoes, anatomical barriers influence viral progression to salivary glands, complicating host infectiousness estimation. We quantified the probability of viral dissemination to the legs in *Ae. albopictus*, which increased with host viral load and was higher after feeding on squirrel monkeys than on cynomolgus macaques. We also found a positive relationship between virus titre in mosquito legs and virus detection in saliva after a 14-day extrinsic incubation period. Combining these factors, we found that squirrel monkeys were on average 1.5 times more infectious to *Ae. albopictus* than cynomolgus macaques. These estimates will help assess ZIKV’s potential to establish an enzootic, sylvatic cycle in the Americas.

## 1. Introduction

Understanding the factors that influence pathogen transmission is essential for predicting and controlling the spread of infectious diseases. In particular, estimating infectiousness—the probability that an infected host transmits a pathogen—is crucial for developing accurate epidemiological models [1]. Modelling at the within-host scale has been employed to derive infectiousness estimates of SARS-CoV-2 and influenza in humans [2], foot-and-mouth disease virus in cattle [3], influenza virus in pigs [3], among others. For vector-borne pathogens, the infectiousness of one host to the next also depends on the vector that bridges the two hosts, as different vector taxa can vary in their competence. Infectiousness estimates exist for dengue virus (DENV) from humans to *Aedes aegypti* mosquitoes [4], Rift Valley fever virus from cattle, sheep and goats to *Aedes* spp. and *Culex* spp. mosquitoes [5], Usutu virus from birds to *Culex quinquefasciatus* mosquitoes [6] and Zika virus (ZIKV) from humans to *Aedes albopictus* mosquitoes [7].

For mosquito-borne viruses, it is commonly assumed that within-host viral load is positively related to the probability of host-to-vector transmission [8–10], although some counter examples have been observed (e.g. DENV in [11]). In the vector, a virus must overcome sequential anatomic barriers (i.e. the midgut infection barrier, the midgut escape barrier and the salivary gland infection and escape barrier) before becoming infectious [12,13]. Experimental constraints can make it difficult to gather enough information to characterize host infectiousness, in the sense of their ability to generate saliva-positive vectors over the complete course of host infection. Hence, existing estimates of host infectiousness are often limited to vector infection or disseminated infection [4,5,7]. Besides, transmission experiments sometimes deliver virus to hosts via needle rather than the bite of an infected vector [5,6], or use artificial blood meals to infect vectors [7], which makes their infectiousness estimates less reflective of the natural transmission arc.

The current study focuses on ZIKV, which originated in a sylvatic cycle involving non-human primate (NHP) hosts and arboreal *Aedes* mosquitoes in Africa [14,15]. It eventually spilled over into humans, establishing human-endemic transmission in the paleotropics mediated by *Ae. aegypti* and *Ae. albopictus* [16]. In addition to its role in epidemics, *Ae. albopictus* may also contribute to both spillover and spillback, as a bridge vector [17,18]. ZIKV reached the Americas early in the 2010s, resulting in widespread human infections [19]. It is not yet known whether the virus will establish new sylvatic cycles within American NHP populations [15,20,21]. Should it do so, control of ZIKV in the Americas will become more complicated and eradication will likely become impossible. In this context, it is crucial to explore the potential for novel monkey hosts to sustain ZIKV transmission. To this end, we aimed to compare the infectiousness of native and novel monkey host species, which results from the coupling between their within-host viral dynamics and the associated transmission to *Ae. albopictus*, following experimental infection with ZIKV strains derived from sylvatic [11] and human-endemic cycles (this paper).

Here, we used these data for phenomenological modelling at the within-host scale to characterize viral load dynamics of ZIKV, focusing on the impact of monkey species, viral strain, initial viral dose received and inter-individual heterogeneity. We then described host-to-vector transmission occurring during those experimental infections, using dose–response relationships, for disseminated infection and saliva infection of *Ae. albopictus*. Finally, we estimated the infectiousness profile of native and novel monkey hosts over the course of their infection, which can readily be used by models of ZIKV transmission dynamics at the population scale.

## 2. Material and methods

### 2.1. Ethics statement

Our study complies with all relevant ethical guidelines and all community standards for containment of infected arthropod vectors; all procedures conducted on NHPs were approved via UTMB Institutional Animal Care and Use Committee (IACUC) protocol 1912100, approved on 1 December 2019.

### 2.2. Overview of the experiment

**Figure 1.**
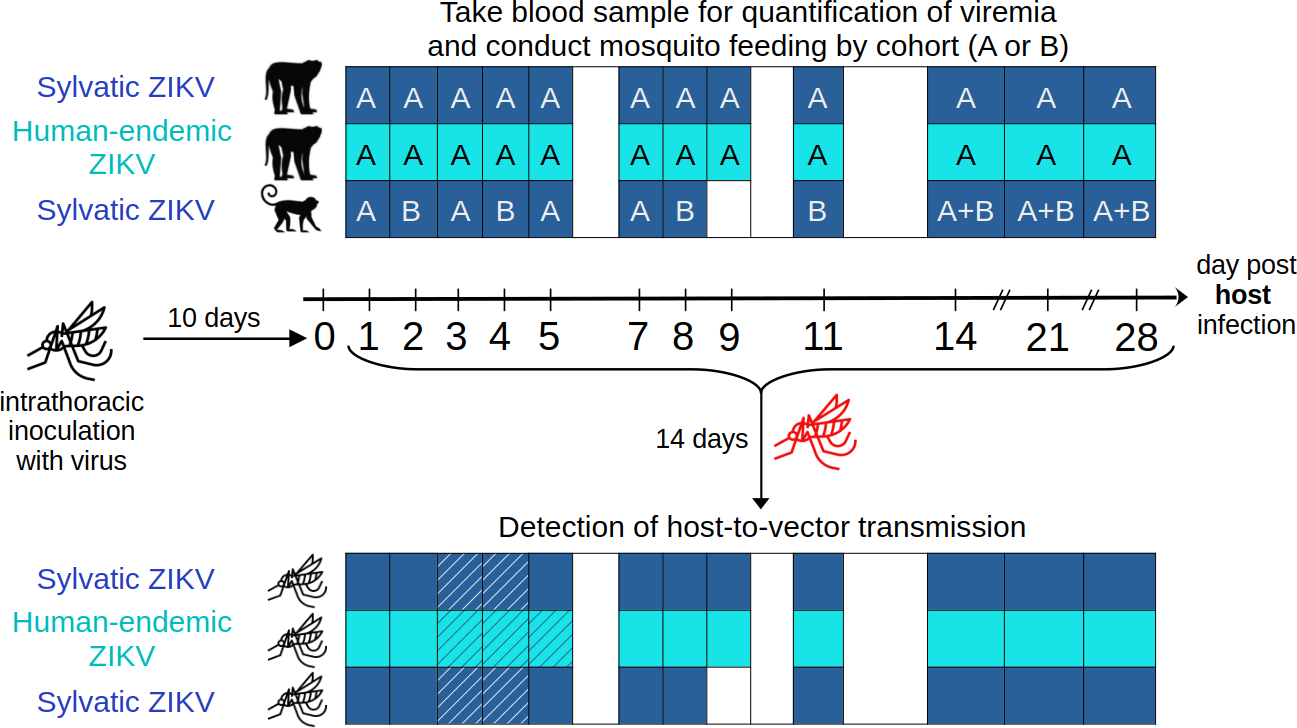
Experimental infections of cynomolgus macaques and squirrel monkeys with sylvatic (DakAr 41525) or human-endemic (PRVABC59) ZIKV. Monkeys were infected through mosquito bite on day 0. Days on the central arrow are all possible sampling days, while the exact days when sampling happened per experimental group are indicated by colour filling in the top grid (dark blue for sylvatic ZIKV, cyan for human-endemic ZIKV, white is not sampled). In the bottom grid, colour filling indicates testing of virus presence in mosquito legs (dark blue for sylvatic ZIKV, cyan for human-endemic ZIKV, white is not sampled) and hatching indicates testing of virus presence in mosquito saliva in addition to legs. The monkey images are licensed from Shutterstock. The mosquito image was free from Flaticon (see Credits section in electronic supplementary material).

Here, we leverage data from previous experimental infections of 3 (1 M, 2 F) adult cynomolgus macaques (*Macaca fascicularis*) and 10 (5 M, 5 F) adult squirrel monkeys (*Saimiri boliviensis*) with sylvatic ZIKV strain DakAr 41525 (GenBank accession number EF105379.1) delivered by the bites of batches of 15 *Ae. albopictus* mosquitoes, described in Hanley *et al.* [11] (figure 1). Infecting mosquitoes had been inoculated intrathoracically with the virus [11]. We also leverage data from another arm of the study (figure 1), not previously reported, in which four adult cynomolgus macaques (two males, weighing 4.7 and 5.2 kg at the start of the experiment, and two females, 2.8 and 2.9 kg at the start of the experiment) were infected using identical methods with human-endemic ZIKV strain PRVABC59 (passaged six times in Vero cells, GenBank accession number KU501215). The number of mosquitoes salivating virus and the total dose of virus delivered were estimated by forced salivation conducted 2 days after infected mosquitoes fed on monkeys; the values for ZIKV DakAr 41525 and PRVABC59 can be found in electronic supplementary material, table S1.

Quantification of viremia from serum as well as feeding of uninfected *Ae. albopictus* mosquitoes on each animal was done regularly between infection and 28 days post-infection (dpi) [11] (figure 1). Viral load was measured by serial dilution of sera which were used to infect Vero cells (CCL-81), followed by immunostaining to yield measurements in plaque-forming units (PFU) [11]. Engorged mosquitoes from all feeding days were tested for the presence of virus in their bodies and legs, 14 days after feeding (single extrinsic incubation period (EIP) tested), as described in Hanley *et al.* [11]. Mosquito saliva was collected and tested only for those mosquitoes that fed on days 3 and 4 (sylvatic strain) or 3, 4 and 5 (human-endemic strain). Table 1 summarizes the number of mosquitoes tested per dpi and per group.

**Table 1.** Mosquitoes tested to detect host-to-vector transmission. *n*, number of monkeys per group. *Five per two cohorts, generally sampled on alternate days, two monkeys had to be euthanized after day 14. Bold values indicate mosquitoes for which both legs and saliva were tested. dpi, days post infection.

monkey	Zika virus	*n*	number of mosquitoes tested per dpi											
species	strain		1	2	3	4	5	7	8	9	11	14	21	28
cynomolgus macaques	sylvatic	3	34	26	**34**	**31**	30	26	37	29	40	21	24	35
cynomolgus macaques	human-endemic	3	34	28	**30**	**32**	**29**	18	26	26	30	23	22	31
squirrel monkeys	sylvatic	10*	44	40	**34**	**21**	59	28	35		35	82	55	73

The 14 days EIP was chosen to maximize the capacity to detect mosquito infection, and limit cases where the virus would not have yet disseminated or reached the saliva at the time of testing. This should not be considered an estimate of the natural EIP of ZIKV in *Ae. albopictus*, which can be assessed in vector competence studies by testing mosquitoes at different times post-feeding [22,23], which was out of the scope of our experiments. The data we present are therefore a static view of mosquito infection at 14 days post-feeding, and do not convey information on within-vector viral dynamics.

The viremia of monkeys over time can be found in electronic supplementary material, table S2, for the ZIKV sylvatic strain and electronic supplementary material, table S3, for the ZIKV human-endemic strain. Of the four cynomolgus macaques infected with human-endemic ZIKV, one (FR1565, see electronic supplementary material, table S3) is excluded from the analyses presented in this article as it never became detectably viremic nor transmitted to mosquitoes. This monkey was fed upon by 83 mosquitoes in total, all of which were negative for virus in every tissue sampled, and these data are also excluded from table 1. Data on transmission to mosquitoes can be found in electronic supplementary material, table S4, for ZIKV sylvatic strain only. Indeed, we only detected one disseminated infection in mosquitoes for the human-endemic strain of ZIKV, and no positive saliva, so we did not analyse this dyad further for host infectiousness.

### 2.3. Within-host viral dynamics

To describe within-host viral dynamics, we used a phenomenological model adapted to capture the pattern of acute, short-lived infections, which relied on the following simple equation [3,24]:


(2.1) $$V(t)=\frac{2.10^{V_{p}}}{e^{-l_{g}(t-T_{p})}+e^{l_{d}(t-T_{p})}},$$

where viral load V at time t is expressed as a function of peak viral load $V_{\text{p}}$ on a log_10_ scale, time of peak $T_{\text{p}}$ and rates of the exponential growth ($l_{\text{g}}$) and decay ($l_{\text{d}}$) phases. We fitted this equation to viral load data from all monkeys simultaneously, using a nonlinear mixed effect modelling approach. We first allowed all four parameters to vary with monkey species, viral strain and dose delivered to the monkeys (fixed effects on three covariates) as well as between individuals (random effects). We then used a forward procedure to select the most relevant covariates and random effects, starting from an empty model. We used the stochastic approximation expectation maximization (SAEM) algorithm, implemented in the R package saemix, v. 3.3 [25]. To ensure that parameters remained positive, we assumed a log-normal distribution, meaning that parameters $\theta_{i}$ of individual i can be expressed as $\theta .e^{f_{i}}$, with θ representing the population-level estimate, and $f_{i}=c_{i}.\beta +\eta_{i}$, with $c_{i}$ representing the subject-specific covariates, β the fixed effects and $\eta_{i}$ the random effects, which follow a multinormal distribution η∼N(0,ω). In addition, we used a constant residual error model. Data below the limit of detection (LOD = 20 PFU ml^−1^) of the assay were treated as left-censored data. Further details about the fitting procedure and the computation of uncertainty can be found in electronic supplementary material, text S1.

### 2.4. Vector infection

As mosquito saliva was only tested on a subset of days, we could not estimate host infectiousness over their whole infection period in a single step. Instead, we first analysed the drivers influencing ZIKV disseminated infection in mosquitoes, as these data were available for all engorged mosquitoes across the entire experiment. We then explored the relationship between disseminated infection and ZIKV presence in mosquito saliva, to ultimately infer the whole dynamics of host infectiousness. For this analysis, host viremia data below the LOD of the assay were set arbitrarily as half the LOD if transmission to mosquitoes was detected, 0 otherwise.

#### 2.4.1. Disseminated infection

We investigated the combined effects of host viral load, host species and dpi on the probability of disseminated infection in vectors ($P_{\text{leg}}$). This probability was defined as the ratio of virus-positive legs over the total number of mosquitoes tested. Sometimes, the ratio of virus-positive legs over the number of positive mosquito bodies is used to measure dissemination [26], but in our case, an important proportion of mosquito legs were positive when the corresponding bodies were negative [11], which made this ratio uninformative. We performed a model selection procedure similar to what was conducted by Lambrechts *et al.* [27], to allow nonlinear effects (electronic supplementary material, text S2.1). A similar approach was used to model the value of the virus titre measured in mosquito legs ($V_{\text{leg}}$).

#### 2.4.2. Probability of ZIKV presence in saliva

We then explored the relationship between $V_{\text{leg}}$ and the probability of ZIKV being detected in that mosquito’s saliva ($P_{\text{saliva}}$), both measured after the same EIP. To do so, we selected among two functional forms with a sigmoidal shape, and between the use of a binomial or a betabinomial likelihood, the latter accounting for overdispersion in the data (electronic supplementary material, text S2.2). The selection was done based on the corrected Akaike information criterion (AICc; the lowest AICc was selected).

### 2.5. Host infectiousness over time

We expressed host infectiousness over time as variations of the transmission rate which, in vector competence studies, is defined as the proportion of saliva-positive mosquitoes among all mosquitoes tested. To do so, we combined the different steps presented above, with the associated uncertainties, as presented in electronic supplementary material, figure S1.

## 3. Results

### 3.1. Within-host viral dynamics

For the full model including the effect of three covariates (monkey species, viral strain, dose delivered) on four parameters ($V_{\text{p}}$, $T_{\text{p}}$, $l_{\text{g}}$, $l_{\text{d}}$; equation (2.1)), as well as random effects on four parameters, the relative standard errors around estimates were large (electronic supplementary material, table S5), indicating an over-parametrization of the model with regard to the quantity of data available.

**Figure 2.**
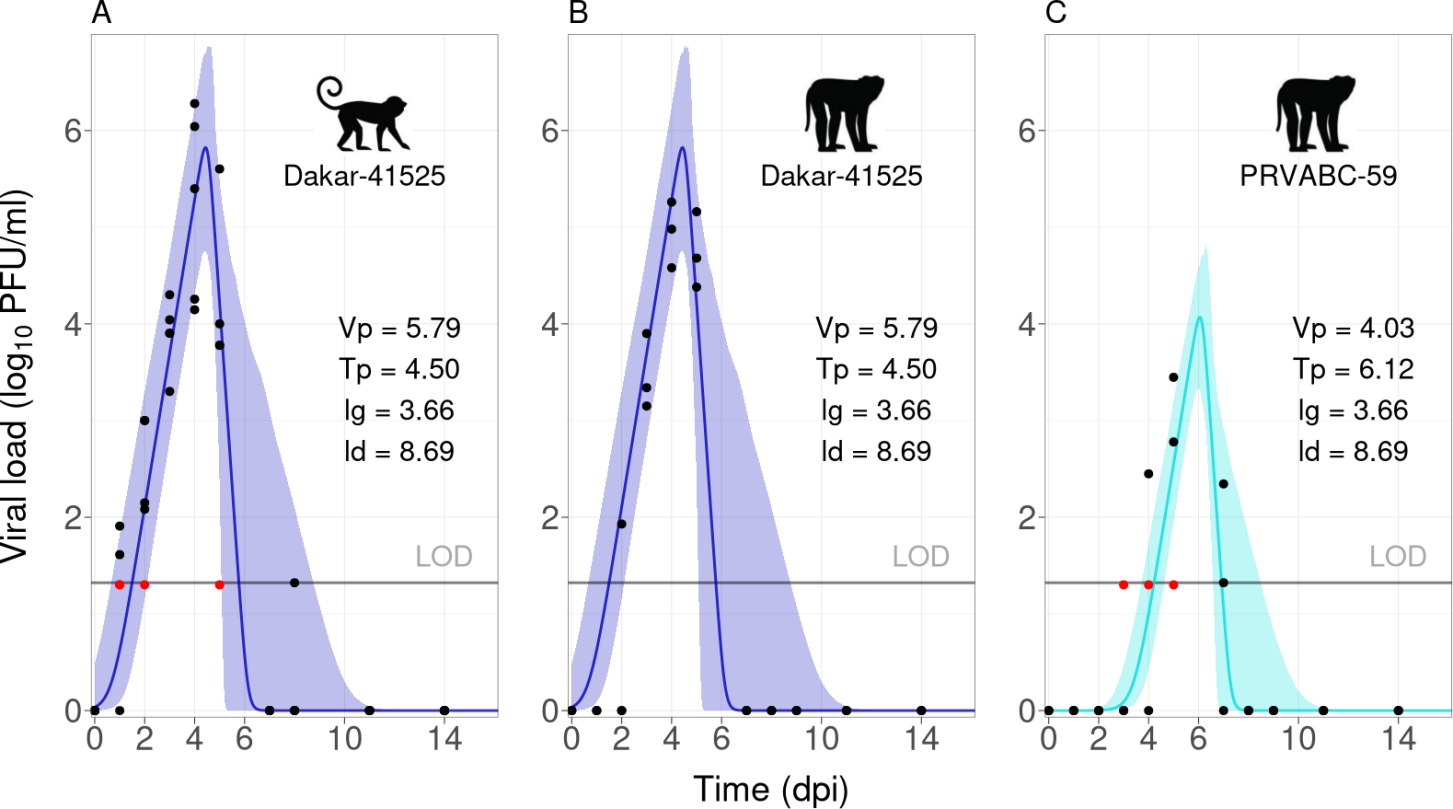
Data and model fit for within-host viral dynamics. (A) Squirrel monkeys infected with sylvatic ZIKV. (B) Cynomolgus macaques infected with sylvatic ZIKV. (C) Cynomolgus macaques infected with human-endemic ZIKV. Group-level model fit (line) and associated parameters. The shaded band is the confidence interval of the fixed effects. Points are raw data (red = left-censored). As no transmission to mosquitoes was detected with the human-endemic strain in cynomolgus macaques, we do not analyse this dyad further for host infectiousness. LOD, limit of detection.

Forward selection retained the effect of viral strain on $V_{\text{p}}$ and $T_{\text{p}}$, and random effects on $V_{\text{p}}$, $l_{\text{g}}$ and $l_{\text{d}}$ (table 2). The human-endemic strain of ZIKV induced significantly lower and later peak titres than the sylvatic strain (figure 2). Monkey species was not retained in the model, meaning that similar parameters could describe the viral dynamics of macaques and squirrel monkeys infected with the sylvatic strain (table 2, figure 2A,B).

**Table 2.** Results of the nonlinear mixed-effect model of within-host viral dynamics, obtained after covariate selection through forward procedure. It incorporates the effect of viral strain on $V_{\text{p}}$ and $T_{\text{p}}$ and random effects on $V_{\text{p}}$, $l_{\text{g}}$, $l_{\text{d}}$. Parameter estimates (relative standard error, %) *p-*value (for the strain covariate). Squirrel monkeys infected with the sylvatic strain of ZIKV are taken as the reference. dpi, days post-infection.

parameter	definition (unit)	fixed effect	random effect variance (ω)
$V_{p}$	peak viral load (log${,}_{10}$ PFU ml^−1^)	5.79 (6.12)	0.036 (37.84)
$\beta_{strain,V_{p}}$	effect of strain on $V_{p}$	−0.36 (39.02) 0.01	
$T_{p}$	time of peak viral load (dpi)	4.50 (2.33)	
$\beta_{strain,T_{p}}$	effect of strain on $T_{p}$	0.31 (9.39) < 2.2 × 10^−16^	
$l_{g}$	exponential rate of growth phase (per day)	3.66 (8.42)	0.11 (37.64)
$l_{d}$	exponential rate of decay phase (per day)	8.69 (29.64)	0.20 (47.51)

There was very little variability among the three macaques infected with the sylvatic strain, and more inter-individual variability among the two other groups (electronic supplementary material, figure S2). $l_{\text{d}}$ and $T_{\text{p}}$ were the most and least variable parameters, respectively (electronic supplementary material, table S6).

### 3.2. Vector infection

#### 3.2.1. Disseminated infection

**Figure 3.**
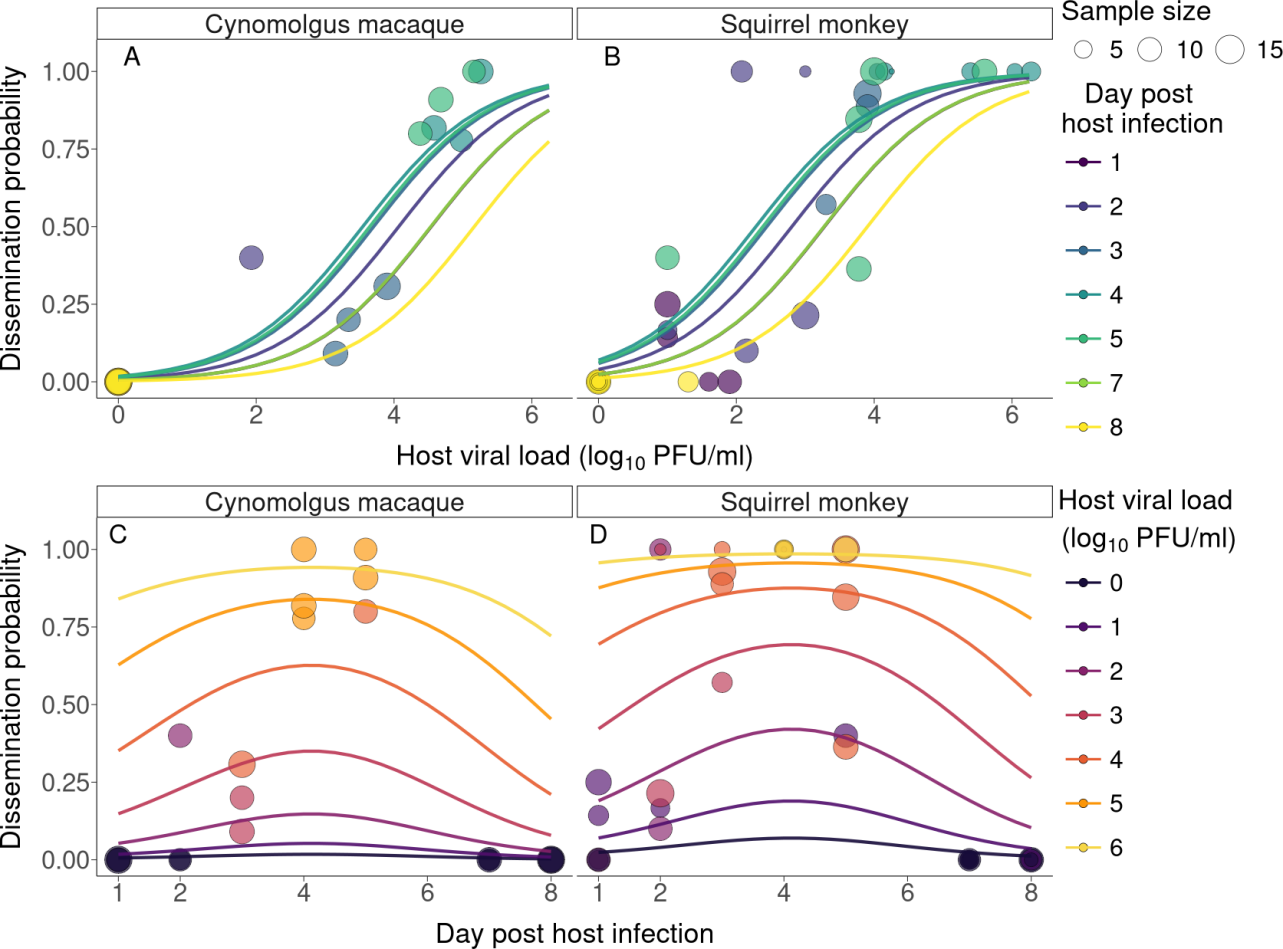
Probability of ZIKV disseminated infection in *Ae. albopictus* as a function of viral load (A,B), day post-infection (C,D) and monkey species (A,C;B,D). Each circle represents a single batch of mosquitoes fed on a given host on a given day, and the size of the circle is proportionate to the number of mosquitoes analysed. Lines represent the regression fits to the data. Lines and data are colour coded according to dpi (A,B) or host viremia (C,D). In (D), note that several points are overlapping for days 4 and 5 and dissemination probability of 1, corresponding to viremia ranging between [4; 6] log_10_ PFU ml^−1^.

The model selected to describe $P_{\text{leg}}$ included a nonlinear, non-decreasing effect of viremia, a nonlinear, non-monotonic effect of dpi and an effect of monkey species (figure 3; electronic supplementary material, text S2.1.1). Viral load had a significant effect (*p* = 9.5 × 10^−12^, figure 3A,B). As an example, at 2 dpi, an increase of viremia from 2.5 to 3.5 log${,}_{10}$ PFU ml^−1^ increases the dissemination probability by 0.27 [0.11; 0.44] after feeding on squirrel monkeys and 0.20 [0.04; 0.36] after feeding on cynomolgus macaques. Monkey species had a significant effect (*p* = 2.5 × 10^−5^), with transmission from squirrel monkeys being more efficient than from cynomolgus macaques (OR = 4.20, 95% CI [2.15; 8.18], figure 3A,B).

Although dpi did not have a significant effect (*p* = 0.065), an interesting pattern emerged, with $P_{leg}$ possibly maximized at 4 dpi even when controlling for viremia (figure 3C,D). We note that the significance of dpi varied from one model to the next in our selection process, and even among models with a nonlinear, bell-shaped effect of dpi, 2 out of 4 models tested estimated the effect of dpi to be significant.

A simple linear model was selected to describe $V_{leg}$ as a function of host viremia, dpi and monkey species (electronic supplementary material, text S2.1.2). Viremia was the only variable with a significant effect (*p* = 7.6 × 10^−10^). We noticed a strong heterogeneity of leg titres, particularly when host viral load was between 4 and 5 log_10_ PFU ml^−1^, for both monkey species (electronic supplementary material, figure S3A). This heterogeneity would not be sufficiently captured in the selected model, as viremia only explained 25% of the variance. To remain simple, for the estimation of host infectiousness, we decided to sample $V_{leg}$ in the whole distribution of leg titres obtained in our experiment. This distribution was bimodal, with frequent sampling around 2.5 and 5.7 log_10_ PFU ml^−1^ (electronic supplementary material, figure S3B).

#### 3.2.2. Probability of ZIKV presence in saliva

**Figure 4.**
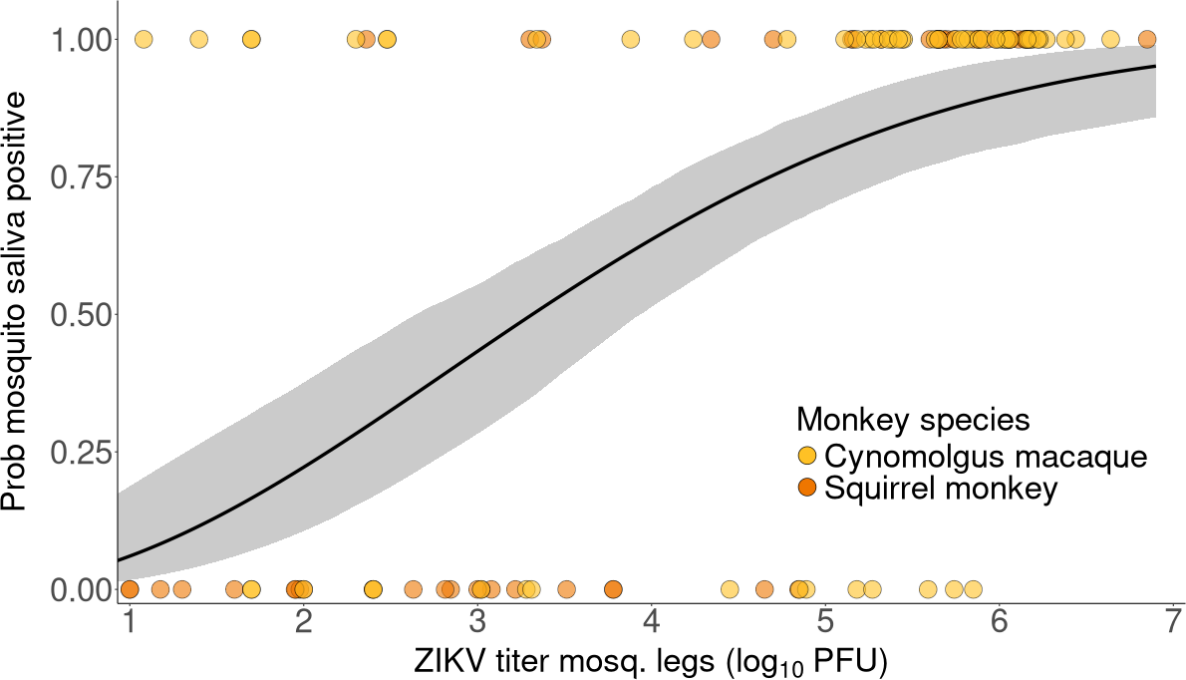
Relationship between ZIKV titre measured in mosquito legs, and probability of mosquito saliva testing positive for ZIKV. Points show raw data, coloured by monkey species, each point representing a single mosquito. The line shows the best fit, obtained with electronic supplementary material, equation (S1), and a binomial likelihood. The shaded band shows the associated 95% confidence interval.

The relationship between $V_{\text{leg}}$ and $P_{\text{saliva}}$ was best described using a Ferguson equation with binomial likelihood (electronic supplementary material, equation (S1)), with $\theta_{0}$= 3.98 [3.35; 4.55] and $\theta_{1}$= 2.01 [1.27; 2.88] (figure 4). This relationship corresponds to mosquito infections arising from both monkey species. Since the distribution of leg titres was similar regardless of the transmitting species (electronic supplementary material, figure S3C), we fitted the relationship for both species combined.

### 3.3. Host infectiousness over time

**Figure 5.**
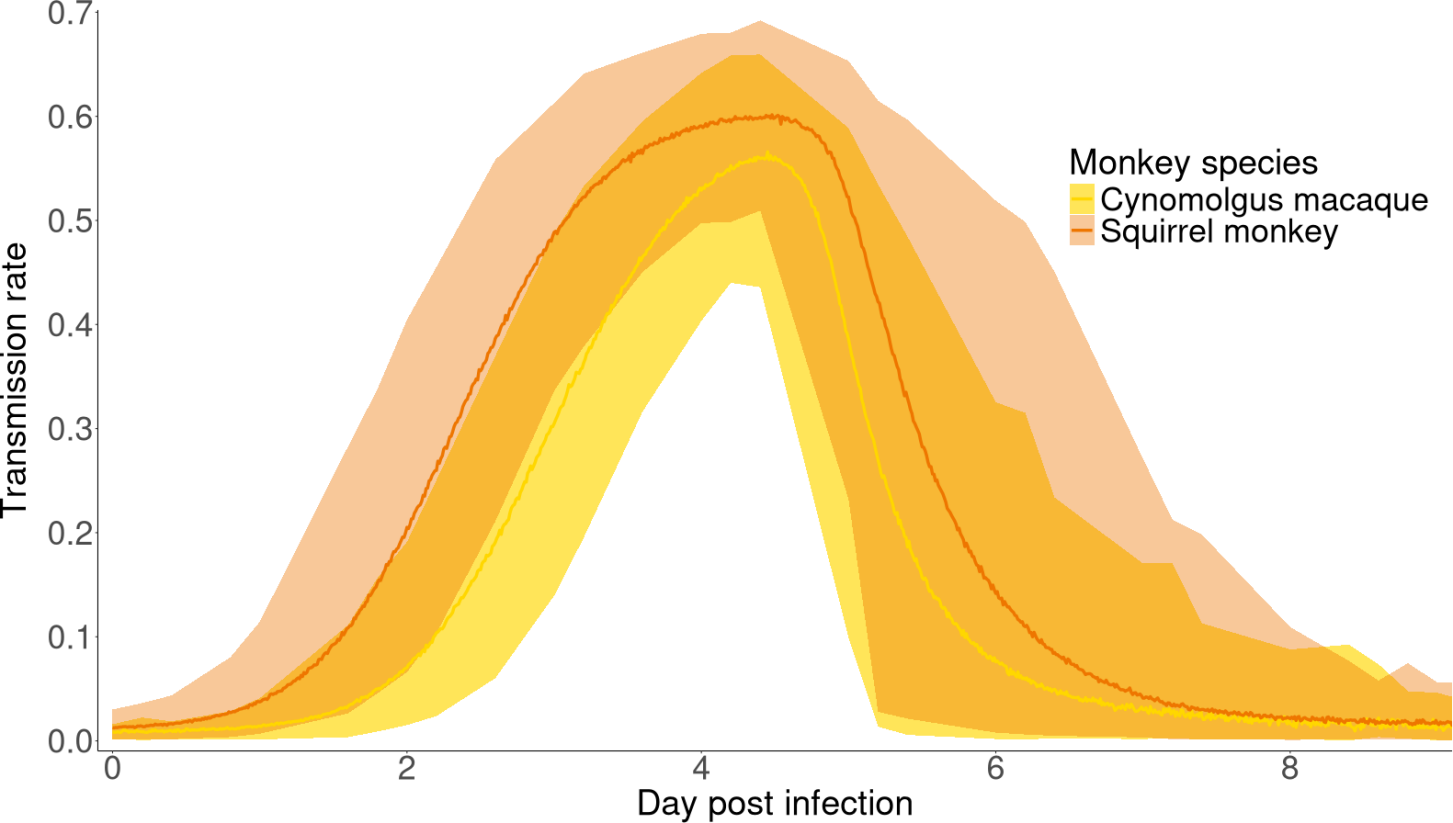
Infectiousness of squirrel monkeys and cynomolgus macaques towards *Ae. albopictus* over time. Lines show the mean transmission rate (probability of mosquito saliva testing positive for ZIKV over the total number of mosquitoes fed) and shaded bands are the associated 95% confidence interval.

The combination of the different steps resulted in the estimation of transmission rates in *Ae. albopictus* over time when feeding on each monkey species infected with sylvatic ZIKV. Squirrel monkeys were on average more infectious than cynomolgus macaques (figure 5), as a consequence of more efficient dissemination in *Ae. albopictus* after feeding on squirrel monkeys than cynomolgus macaques.

Comparing the area under the curve (AUC) of their infectiousness profiles, we estimated that squirrel monkeys were on average 1.46 times more infectious (95% confidence interval [1.36; 1.56]) than cynomolgus macaques. This is assuming both species are subject to similar contact rates with vectors. The maximum transmission rate was similar for both species (mean 0.57 [0.46; 0.67] for cynomolgus macaques, 0.60 [0.51; 0.69] for squirrel monkeys, figure 5), due to a common sampling distribution for $V_{\text{leg}}$ and $P_{\text{saliva}}$. This maximum was reached at 4 dpi (figure 5). As a conservative estimate, we also derived the infectiousness profiles assuming viral loads below LOD would not give rise to any infection (electronic supplementary material, figure S4). The ratio of AUC between species remained similar (1.43 [1.33; 1.52]).

## 4. Discussion

In this study, we leveraged data from an experiment coupling ZIKV infection of monkeys with transmission to *Ae. albopictus* vectors to derive estimates of monkey infectiousness over time. The experiment was replicated across two species of monkey, one (cynomolgus macaque) a native host of the virus, and another (squirrel monkey) a novel host with potential to serve as a New World reservoir in the future, as well as two strains of the virus, one each from the sylvatic and human-endemic ecotype [28]. We found that monkey species had no significant effect on within-host viral dynamics, while virus strain did, with the human-endemic strain inducing later and lower viremia peaks than the sylvatic strain. Moreover, the human-endemic strain failed to transmit to *Ae. albopictus* mosquitoes, but the sylvatic strain robustly transmitted. For the sylvatic strain, host viral load and host species both influenced disseminated infection in mosquitoes ($P_{\text{leg}}$), with more efficient transmission at higher viremia, and from squirrel monkeys than cynomolgus macaques. Turning to infectious mosquitoes, we found a positive relationship between $V_{\text{leg}}$ and $P_{\text{saliva}}$ after an EIP of 14 days. Taken together, these results allowed us to conclude that squirrel monkeys were overall 1.5 times more infectious than cynomolgus macaques towards *Ae. albopictus*, when infected with a sylvatic strain of ZIKV, while the trend in human-endemic ZIKV could not be investigated due to a dearth of transmission. However, this conclusion assumes equal contact of both host species with mosquitoes, which is questionable, as we have recently shown that *Ae. albopictus* engorged more frequently on cynomolgus macaques than squirrel monkeys, in this same experiment [29]. Further investigations should assess to what extent the interaction between host competence and vector trophic preferences can shape the contribution of different host taxa to transmission [30–32].

The transmission rates estimated here can readily be used to improve population-level models, such as that of Althouse *et al.* [20]. This underscores the utility of the framework we have developed, which exploits all aspects of the experimental design to provide an accurate picture of monkey infectiousness over time. Indeed, if we had only focused on within-host viral dynamics, we would have concluded that controlling for contact rates with vectors, squirrel monkeys and cynomolgus macaques were likely to infect mosquitoes similarly, which would have been a mistake. Of note, quantifying viral titre in mosquito legs was instrumental for us to link the different components of infectiousness, but this type of measure is not done systematically in vector competence studies. In the future, our framework can easily be applied to similar experiments involving other arbovirus, hosts, or vectors.

The modelling techniques we used were adapted to the quantity of data available, but do not provide mechanistic insight to explain observed differences between viral strains and monkey species. The equation used to describe within-host viral dynamics has the advantage of only having four parameters, which can all be estimated at the individual and population levels. In comparison, even simple, target-cell limited, mechanistic within-host models, with no compartments for the immune response, which have been used in previous studies of within-host ZIKV dynamics [7,33–35], have four to five parameters and two initial conditions. When these are fitted to datasets of viral loads over time, some of these parameters and initial conditions must be fixed, sometimes arbitrarily or with the help of the literature, which can limit the mechanistic insight gained. When the objective is to capture the overall dynamics to accurately estimate host infectiousness over time, as was the case here, phenomenological modelling is sufficient and easier to implement. If one wishes to dive deeper into the processes of host–pathogen interactions, to explore different immune mechanisms or the effect of treatment, the mechanistic approach may be better suited. This endeavour will however require fitting to data other than viral loads alone [24,36]. Regarding mosquito infection, the development of a dynamic mechanistic model was not possible as we only had data for a single EIP. Acquiring data at different EIPs usually requires sacrificing mosquitoes, which needs to be accounted for in models and also limits the number of individuals tested per time step. To bypass this issue, possible alternatives consist of monitoring viral shedding in mosquito excreta [37] or having live mosquitoes salivate on sugar pads [38].

The human-endemic ZIKV strain used here (PRVABC59) has been used in previous experimental infections of monkeys, albeit comparisons with our study are limited by differences in monkey species used (rhesus macaques *Macaca mulatta* in [33,35,39–43], cynomolgus macaques in [44,45]), inoculation route (subcutaneous in all except Dudley *et al.* [39] who used both mosquito bite and needle delivery) or type of viremia measured (genome copies in all except Triplett *et al.* [40]). Dudley *et al.* [39] used mosquito (*Ae. aegypti*) bites to deliver PRVABC59 to rhesus macaques, and measured viremia via qRT-PCR. Similar to our results, they observed that viremia peaked at 5 or 6 dpi. In Dudley *et al.*, however, viremia was detected earlier (from 1 dpi for three or four individuals versus never before 3 dpi in our case) and lasted longer (at least until day 8 versus never after day 7 in our case). This likely reflects differences in detectability and persistence between viral genome copies and infectious virus. Indeed, in Triplett *et al.* [40] infectious viremia peaked at 2.5−3 log_10_ PFU ml^−1^ in rhesus macaques, similar to our study, and was resolved by day 5. Dudley *et al.* noted that peak viremia was reached 1−2 days later with mosquito-bite inoculation compared to subcutaneous inoculation. We observe the same delay when comparing our results to studies subcutaneously inoculating doses between 3 and 4 log_10_ PFU ZIKV [33,35,43,45], which is similar to the dose we estimated was delivered by mosquitoes in the current experiment (min.–max. [2.80−4.47] log_10_ PFU ZIKV). Studies using a sylvatic strain closely related to the one we used (Dakar 41524) all infected pregnant rhesus macaques subcutaneously, and measured viremia through genome copies [43,46,47]. Interestingly, in a subset of these, Crooks *et al.* [43] were able to compare viral dynamics between strain PRVABC59 and Dakar 51424 and observed later and lower peak viremia for PRVABC59, similar to what we found, although these differences were not significant in their case.

Regarding the drivers of host-to-vector transmission, we observed a pattern similar to what was recently reported by Lambrechts *et al.* [27], who found maximum transmission of DENV from humans to *Ae. aegypti* at day 2 post-symptom onset, after controlling for levels of viremia. In our case, maximum transmission was reached at 4 dpi, but this association was not significant in the final selected model. The fact that this pattern emerged in our study, where we measured infectious viremia, compared to the data in Lambrechts *et al.*, who measured genome copies, suggests that infectivity and therefore detection of viral particles *per se*, as might be caused by attachment of host antibodies that interfere with an infection assay, is not responsible for this difference in transmission between early and late infection. The mechanistic basis of this phenomenon remains to be elucidated.

The likelihood of ZIKV establishing a sylvatic transmission cycle in the Neotropics cannot be inferred from our data alone. Compelling evidence of the existence of a ZIKV sylvatic cycle would include: (i) experimental demonstration that wildlife–mosquito–wildlife transmission can be sustained; (ii) detection of epizootics in sylvatic settings isolated from spillover from the human-endemic cycle; and (iii) genetic divergence of ZIKV strains isolated from wildlife and/or mosquitoes during epizootics from strains isolated from human transmission cycles. To date, at least one study has reported circulation of African lineage ZIKV in Brazil [48], and sylvatic DENV has been transported multiple times out of both Africa and Asia to other continents [49–51], demonstrating the potential for sylvatic ZIKV to be introduced to the Americas. With regard to experimental evidence of sustainable transmission, the sylvatic strain we tested transmitted efficiently between squirrel monkeys and *Ae. albopictus*, but this is only one host–vector combination out of many possible in the field. The human-endemic strain tested here belongs to the clade circulating in the New World (American lineage [52]), and did not transmit well to NHPs in the present study. While carcasses of free-living marmosets (*Callithrix* sp.) from Brazil have been tested positive for ZIKV by PCR, with a strain belonging to the American lineage [21], this finding may represent spillover from the human-endemic cycle rather than an epizootic. Host preferences will also shape potential for sustained transmission, and as noted above, we have shown that *Ae. albopictus* engorged at higher rates on cynomolgus macaques compared to squirrel monkeys, even when these mosquitoes were not given a choice between host species [29]. To move forward in understanding the potential for ZIKV to establish a sylvatic cycle in the Americas, the co-occurrence of vectors and hosts must be mapped at high granularity, and mosquito blood meals must be analysed to identify which vectors competent for ZIKV feed on both humans and NHPs and the frequency at which they do so, and viruses must be isolated from wildlife and sylvatic mosquitoes to discern nascent sylvatic lineages. Such studies would need to be stratified vertically, as the community composition at ground level can greatly differ from that at canopy level [53,54].

## Data Availability

The datasets supporting this article have been uploaded as part of the electronic supplementary material. The code to reproduce all analyses is available in the Zenodo repository [55]. Electronic supplementary material is available online [56].
